# Effects of novel organic sources of cobalt on ruminal fermentation, nutrient degradation and vitamin B_12_ synthesis in vitro

**DOI:** 10.1093/tas/txaf123

**Published:** 2025-09-17

**Authors:** Jose A Arce-Cordero, Martha U Siregar, Gerald K Salas-Solis, Ana C Silva Vicente, James R Vinyard, Efstathios Sarmikasoglou, Mikayla L Johnson, Richard R Lobo, Szu W Ma, Charese Hammond, Kaliandra S Alves, Michael Socha, Antonio P Faciola

**Affiliations:** Escuela de Zootecnia, Universidad de Costa Rica, San Jose 11501-2060, Costa Rica; Department of Animal Sciences, University of Florida, Gainesville, FL 32611, United States; Department of Animal Sciences, University of Florida, Gainesville, FL 32611, United States; Department of Animal Sciences, University of Florida, Gainesville, FL 32611, United States; Matanuska Experiment Farm and Extension Center, University of Alaska Fairbanks, Palmer, AK 99645, United States; Department of Animal Sciences, Michigan State University, East Lansing, MI 48824, United States; Department of Animal Sciences, University of Florida, Gainesville, FL 32611, United States; Department of Animal Sciences, University of Florida, Gainesville, FL 32611, United States; Department of Animal Sciences, University of Florida, Gainesville, FL 32611, United States; Department of Animal Sciences, University of Florida, Gainesville, FL 32611, United States; Department of Animal Sciences, University of Florida, Gainesville, FL 32611, United States; Research and Discovery, Zinpro Corporation USA LLC, Eden Prairie, MN 55344, United States; Escuela de Zootecnia, Universidad de Costa Rica, San Jose 11501-2060, Costa Rica

**Keywords:** cobalamin, cobalt amino acid, cobalt carbonate, cobalt pectin

## Abstract

Cobalt (Co) is essential for vitamin B_12_ synthesis in the rumen and energy metabolism in cattle. Previous studies on organic Co sources have shown variable effects on dairy cows’ performance, focusing primarily on Co glucoheptonate as an alternative to the traditional Co carbonate (CoCO_3_). Our objective was to evaluate the effects of novel organic Co sources on ruminal microbial fermentation, nutrient degradation, and vitamin B_12_ synthesis using a dual-flow continuous culture system. We used eight fermenters in a duplicated 4 × 4 Latin Square design, where each ­fermenter was provided daily with 106 g DM of a basal diet plus the corresponding Co treatment. The treatments consisted of supplementing 1 mg Co/kg DM from the following Co sources: (i) CoCO_3_, (ii) Co Pectin (CoPec), (iii) Co Amino Acid (CoAA), and (iv) Co Pectin + Co Amino Acid (CoPec+CoAA). Data were analyzed with the MIXED procedure of SAS 9.4 to evaluate the effect of Co source on pH, VFA, N metabolism, vitamin B_12_ concentration and flows, and nutrient degradability. We did not find an effect of Co source on pH (*P *= 0.92) or concentrations of acetate (*P *= 0.32), propionate (*P *= 0.15), butyrate (*P *= 0.92), and other VFA. However, we found that D-lactate concentration was greater in CoCO_3_ than other treatments (*P *= 0.03), and total lactate was greater in CoCO_3_ compared to CoAA and CoPec+CoAA (*P *= 0.04). Vitamin B_12_ in liquid outflow tended to be lower with CoCO_3_ (*P *= 0.10). Moreover, there was greater bacterial N flow (*P *= 0.01) and also greater N use efficiency (*P *= 0.01) in CoAA than CoCO_3_ and CoPec. For nutrient degradation, we found a greater ruminal degradability of NDF (*P *< 0.01) in CoAA and CoPec+CoAA compared to CoCO_3_ and CoPec. Our results indicate that, at a supplementation rate of 1 mg Co per kg diet DM, the tested Co sources affected fermentation of ruminal contents in continuous culture. Specifically, under the conditions of this experiment with dairy cattle diets, CoAA promoted greater microbial protein synthesis, NDF degradability, and liquid-associated vitamin B_12_ flow compared to CoCO_3_ and CoPec, suggesting enhanced Co utilization by ruminal microorganisms when CoAA is included as the primary Co source in the diet.

## Teaser

Cobalt (Co) is an essential nutrient that plays a key role in ruminal fermentation and energy metabolism of ruminants. We evaluated organic sources of Co which may be more soluble and have a positive impact on ruminal fermentation compared to the traditional source of supplemental Co (CoCO_3_) in dairy cattle diets.

## Introduction

Cobalt (Co) is essential for ruminal microorganisms to synthesize vitamin B_12_ (NRC 2001), which is a critical component of methylmalonyl coenzyme A mutase and methionine synthetase. These enzymes play key roles in propionate and energy metabolism in ruminants (Stemme et al., 2008). When sufficient dietary Co is available, ruminal microorganisms can synthesize vitamin B_12_ in amounts that meet the nutrient requirements of both ruminal microorganisms and the host animal (Casper et al., 2021).

Research on Co supplementation in dairy cows shows variable effects on milk production and vitamin B_12_ status (Kincaid et al., 2003; Kincaid and Socha, 2007; Akins et al., 2013). Current dietary Co supplementation guidelines recommend 0.15 mg Co/kg DM for beef cattle (NASEM 2016) and 0.20 mg Co/kg DM for dairy cows (NASEM 2021). However, actual supplementation concentrations often exceed these recommendations due to potential benefits of greater Co intake, especially when cattle consume large amounts of starch, such as the case of receiving and finishing diets with grain inclusion rates ranging from 20 to 90% of DM, where nutritionists report supplementation of 0.82 to 1.03 mg of Co/kg of diet DM (Samuelson et al., 2016). Research suggests that increasing Co supplementation up to 1.0 mg Co/kg DM may enhance vitamin B_12_ synthesis and status of ruminants (Mills 1981; Tiffany et al., 2006; Kincaid and Socha 2007).

Uncertainty remains regarding whether organic Co sources offer benefits over traditional sources, such as CoCO_3_. Based on multiple regression slope ratios of vitamin B_12_ concentration on added Co, Kawashima et al. (1997) estimated that in vitro relative bioavailability of Co was equivalent to 100, 91, 84, and 0% for Co sulfate, CoCO_3_, Co glucoheptonate, and Co oxide, respectively. Studies in cattle have compared CoCO_3_ with organic sources, finding mixed results that span from similar Co concentrations of tissues and animal performance in animals supplemented with Co acetate or Co lactate with high doses up to 4.8 mg of Co/kg of DM (Raths et al., 2023) to greater ruminal propionate and plasma glucose in animals fed Co propionate at 0.1 mg of Co/kg of DM (Tiffany et al., 2003). Moreover, most studies of organic Co supplementation focus on Co glucoheptonate as an alternative to CoCO_3_ (Kincaid and Socha 2007; Akins et al., 2013; Casper et al., 2021).

This study aimed to evaluate the effects of novel organic Co sources, supplemented at 1.0 mg Co/kg DM in high-producing dairy cow diets, on ruminal microbial fermentation, nutrient degradation, and vitamin B_12_ synthesis using a dual-flow continuous culture system. We hypothesized that different Co sources (CoCO_3_, Co pectin, and Co amino acid) would affect ruminal fermentation differently, with organic Co sources enabling more efficient Co utilization by ruminal microorganisms, and therefore greater synthesis of vitamin B_12_ and propionate compared to CoCO_3_.

## Materials and methods

All animal care and use procedures were approved by the University of Florida Institutional Animal Care and Use Committee (IACUC).

### Experimental design and diets

Nutrient degradation, microbial fermentation, and N metabolism were examined using a dual-flow continuous culture system. Eight fermenters were used in a duplicated 4x4 Latin square design with 4 treatments and 4 fermentation periods. All fermenters received a common basal diet (Table 1) and treatments were defined by the supplemental source of Co as follows: (i) Cobalt Carbonate (**CoCO_3_)**, (ii) Cobalt Pectin (**CoPec), (**iii) Cobalt AA (**CoAA)**, and (iv) a combination of Co AA and Co Pectin (**CoPec+CoAA)**. All treatments received 1.65 mg of total Co per kg of diet DM, which corresponded to 1.0 mg of supplemental Co (from the corresponding source or combination of sources) and 0.65 mg of Co from the basal diet. Organic sources (CoPec and CoAA) are presumably more soluble and available than CoCO_3_, therefore expected to impact fermentation by ruminal microorganisms (ie greater propionate and vitamin B_12_ synthesis). By combining both we could evaluate if there is a synergistic effect when both Co sources are combined in the diet compared to supplementing each source separately.

**Table 1.** Ingredient and chemical composition of experimental diets for dual-flow continuous culture experiment.

		**Treatments** ^a^			
Items^b^		CoCO_3_	CoPec	CoAA	CoPec+CoAA
**Ingredients, % of DM**					
** Corn silage**		42.9	42.9	41.9	42.7
** Grass hay**		15.7	16.0	15.9	15.9
** Ground corn grain**		16.0	16.0	16.5	16.0
**Soybean meal 48%CP**		21.1	21.1	21.1	21.1
** Cobalt premix^c^**		2.52	2.35	2.75	2.49
** Calcium Carbonate**		0.84	0.84	0.84	0.84
** Calcium Phosphate**		0.55	0.55	0.56	0.55
** Magnesium Oxide**		0.33	0.33	0.33	0.33
**Chemical composition, % of DM**					
** CP**		16.3	16.0	16.3	16.30
** NDF**		33.5	33.4	33.4	33.50
** Starch**		30.9	30.9	30.9	30.87
**NEl, Mcal kg^−1^ of DM**		1.52	1.52	1.52	1.52
** Ca**		0.70	0.70	0.70	0.70
** P**		0.40	0.40	0.40	0.40
** Mg**		0.35	0.35	0.35	0.35
**Total Co (mg/kg DM)**	1.65		1.65	1.65	1.65
**Supplemental Co (mg/kg DM)**	1.00		1.00	1.00	1.00

a Experimental treatments: **CoCO_3_**, Cobalt Carbonate; **CoPec**, Co Pectin; **CoAA**, Co Amino Acid; **CoPec+CoAA**, Co Pectin + Co Amino Acid. Supplemental Co sources provided a total of 1 mg Co/kg DM. The sources of Co used for this experiment were provided by Zinpro Corporation USA LLC.

b Items: DM = Dry Matter, CP = Crude Protein, NDF = Neutral Detergent Fiber, NEl = Net Energy for milk production, Ca = Calcium, P = Phosphorus, Mg = Magnesium, Total Co = Milligrams of Co (from premix and basal diet) per kg of total diet DM, Supplemental Co = Milligrams of supplemental Co (from premix) per kg of total diet DM.

c Level of inclusion was defined based on Co concentration, with the objective of providing 1 mg of supplemental Co/Kg diet DM. Concentrations of Co in premixes were as follows: CoCO_3_ (39.92 mg/kg DM), CoPec (42.83 mg/kg DM), CoAA (36.64 mg/kg DM), and CoPec+CoAA (40.45 mg/kg DM).

Two Holstein cows fitted with ruminal cannula were used as the source of ruminal contents for all the experimental periods. Both cows were at the University of Florida’s Dairy Research Unit, were in mid lactation (130 DIM on average) and averaged 680 kg of body weight. From −20 d until the end of the experiment, both cannulated cows were fed ad libitum at 0600 and 1400 h a TMR with the following composition (on a DM basis): 38% corn silage, 19% ground corn, 13% soybean meal, 11% cotton seed, 9% citrus pulp, 8.5% mineral premix, and 1.5% palmitic acid supplement.

Ruminal contents were collected 2 h after morning feeding and strained through four layers of cheesecloth, placed into warm thermos jars, and transported to the lab within 30 min of collection. At the lab, each fermenter jar was filled with a 50:50 mix of ruminal content from the 2 cows. All diets in this experiment were formulated to meet the requirements of a lactating Holstein cow (680 kg) producing 45 kg of milk per day (4.8% lactose, 3.5% fat, and 3.0% protein; NASEM 2021).

Each fermenter was fed 106 g/d of basal diet DM divided equally between 2 feeding times, in the morning and evening (0800 and 1800 h). Main dietary ingredients (corn silage, grass hay, ground corn grain and soybean meal) were dried for 72 h in a forced-air drying oven at 60 °C and then ground to pass a 2 mm screen (Wiley mill model 2; Arthur H. Thomas Co., Philadelphia, PA, United States). Dried and ground ingredients were individually weighed by hand using an analytical scale to prepare the total diet for each fermenter at each feeding time. Treatments consisted of the supplemental source of Co and were randomly assigned within Latin square for each period. Each period consisted of 10 d distributed in 7 d of diet adaptation and 3 d of sample collection.

### Dual-flow continuous culture system

We used a dual-flow continuous-culture system (Hoover et al., 1976) with adaptations described by Paula et al. (2017). In this system, each fermenter was infused with artificial saliva (Weller and Pilgrim 1974) at 3.05 mL/min using a peristaltic pump (Model 07522-20; Masterflex; Gelsenkirchen, Germany) to achieve an 11% h^−1^ liquid dilution rate and 5.5% h^−1^ solid dilution rate (Brandao and Faciola 2019). Contents of the fermenters were maintained at 39 °C with constant agitation at 100 rpm for continuous mixing.

Samples were collected from the effluent containers of each fermenter and from artificial saliva containers on d 5 before infusion of (^15^NH_4_)_2_SO_4_ to determine the basal ^15^N concentration. Then, before morning feeding, each fermenter was infused with a pulse dose of 0.1173 g of ^15^N labeled (^15^NH_4_)_2_SO_4_, and artificial saliva was replaced with artificial saliva containing (^15^NH_4_)_2_SO_4_ to achieve a constant inflow of ^15^N into the fermenters to estimate the microbial uptake of N. The samples collected were freeze-dried, ground with a mortar and pestle, and stored at −20 °C until analyzed for background ^15^N concentration.

### Collection of samples

To evaluate variation in microbial fermentation conditions samples were collected on d 8—10 for pH measurement and analyses of lactate, NH_3_-N, VFA, and vitamin B_12_. The pH of contents in each fermenter was measured using a portable pH probe (Orion Star A121; ThermoFisher Scientific, Waltham, MA). At 3, 6, 9, and 24 h post morning feeding, contents of the solid and liquid effluent containers of each fermenter were mixed, and a sample of approximately 200 g was collected. A daily composite sample was obtained from each fermenter by combining liquid and solid effluents and mixing for 20 s with a hand mixer in a clean container. Composite samples were collected for analyses of nutrient degradation and concentrations of VFA, lactate, vitamin B_12_, and NH_3_-N. Samples for nutrient degradation analysis were collected in duplicate and stored at −20 °C until lyophilized and analyzed for nutrient composition. For analyses of VFA and NH_3_-N concentrations, approximately 12 mL were collected and strained through 4 layers of cheesecloth. A 2 mL aliquot was reserved for lactate and vitamin B_12_ analysis. The remaining 10 mL were mixed with 100 µL of 50% H_2_SO_4_ to stop fermentation and prevent volatilization of NH_3_-N. All samples were immediately frozen and stored at −20 °C.

On the final day of each period (d 10), the whole content of each fermenter was processed by 3 consecutive centrifugations in order to isolate the bacterial pellet (Krizsan et al., 2010). The contents of each fermenter were blended for 30 s, filtered through four layers of cheesecloth, and then washed using 400 mL of 0.9% saline solution. Filtered fermenter contents were then centrifuged at 1,000 × g for 10 min to remove any remaining feed particles. The supernatant from the initial centrifugation was then centrifuged at 11,250 × g for 20 min to isolate the bacterial pellet. The pellet was then resuspended in 200 mL of McDougall’s solution and subsequently centrifuged at 16,250 × g for 15 min. The supernatant was then discarded, and the resulting bacterial pellet was transferred to a plastic container and frozen at −20 °C until lyophilized for further analyses of ^15^N, N, and OM content.

### Calculations for disappearance of nutrients and N metabolism

The different N fractions were estimated for evaluation of N metabolism and true degradability of nutrient fractions. Total N in digesta effluent corresponds to the N remaining after microbial ruminal fermentation, which is divided into the following fractions: NH_3_-N flow, dietary N flow, and microbial N flow. Moreover, the flow of non-ammonia N (NAN) is a combination of dietary N flow and microbial N flow.

The flows of NH_3_-N, NAN, and microbial N were determined according to Calsamiglia et al. (1996) and Bach and Stern (1999), using the following equations:

1. NH_3_-N flow (g/d) = NH_3_-N in effluent (g/dL) × (mL of total effluent flow/100).
2. NAN flow (g/d) = g of total N in effluent − g of effluent NH_3_-N.
3. Microbial N flow (g/d) = (NAN flow × % atom excess of ^15^N in NAN effluent)/(% atom excess of ^15^N in bacteria pellet).
  Where % atom excess of ^15^N in NAN effluent = % atom ^15^N in NAN effluent sample—basal % atom ^15^N.
  Additionally, the flow of dietary N and microbial efficiency indicators were determined according to Bach and Stern (1999) as follows:
4. Dietary N flow (g/d) = g of NAN in effluent − g of bacterial N in effluent.
5. Microbial efficiency = microbial N flow (g)/OM truly digested (kg).
6. Efficiency of N use (ENU) = (g of microbial N/g of available N) × 100.
  Finally, the true ruminal degradability of nutrient fractions (OM, CP, NDF, and starch) was calculated as follows:
7. % Nutrient degradability (DM basis) = 100 × [g of nutrient in diet – (g of nutrient in effluent—g of nutrient in saliva—g of nutrient in microbial mass)]/g of nutrient in diet.

### Chemical analyses

Samples of feed ingredients and freeze-dried digesta were analyzed for DM, using an air-forced oven at 105 °C for 12 h, according to the method 930.15 (AOAC, 1990). Ash was also determined, using a muffle furnace at 600 °C for 6 h, according to the method 942.05 (AOAC, 1990), NDF (Van Soest et al., 1991) adapted for Ankom200 Fiber Analyzer (Ankom Technology, Macedon, NY, United States) with heat-stable α-amylase and sodium sulfite, and total N (AOAC International, 2000; method 990.03).

Acidified rumen fluid samples that were collected for VFA and NH_3_-N concentration analysis were centrifuged (Sorvall LYNX 4000 Centrifuge, Thermo Scientific) at 10,000 × g for 15 min at 4 °C. The NH_3_-N concentration in supernatant was determined in duplicate according to Broderick and Kang (1980) and adapted to a plate reader by using 2 µL of the sample, 100 µL of phenol solution and 80 µL of hypochloride solution in each well of the microplate. Absorbance was measured in a spectrophotometer (SpectraMax Plus 384 Microplate Reader, Molecular Devices) at 620 nm. The inter- and intra-assay coefficient of variability of the analysis was 2.85 and 5.37%, respectively.

The remainder of the supernatant was processed according to Ruiz-Moreno et al. (2015) for the analysis of short-chain fatty acids. Briefly, 2 mL of ruminal fluid supernatant was combined with 0.4 mL of a 5:1 ratio metaphosphoric acid and crotonic acid solution to utilize crotonic acid as an internal standard. Concentrations of acetate, propionate, butyrate, isobutyrate, isovalerate and 2-methylbutyrate, valerate, and caproate in samples were analyzed by GC (Agilent 7820A GC, Agilent Technologies) with a flame ionization detector and a capillary column (CP-WAX 58 FFAP 25 m, 0.53 mm, Varian CP7767, Varian Analytical Instruments) at 110 °C with injector temperature at 200 °C and detector at 220 °C.

Lactate concentration was determined using a commercial kit (D-Lactic acid/L-Lactic acid kit, R-Biopharm AG). Absorbance was measured in a spectrophotometer (SpectraMax Plus 384 Microplate Reader, Molecular Devices). The inter- and intra-assay CV of the analysis were 8.87 and 5.17%, respectively.

Based on expected ^15^N concentrations, 1, 2, or 4 mg of bead-beaten sample (for samples of bacteria, digesta effluent, and digesta ^15^N background, respectively) were loaded into an 8 × 5 mm tin capsule (Elemental Microanalysis) using a Mettler-Toledo Excellence Plus XP Micro Balance (Mettler-Toledo GmbH, Laboratory and Weighing Technologies). To evaporate NH_3_-N in the samples, 35 µL of 10 g/L K_2_CO_3_ was added to each sample and dried overnight in a forced-air oven at 40 °C according to Schwab et al. (2006). Then, % ^15^N in dried samples was analyzed in an isotope ratio mass spectrometer (IsoPrime 100, IsoPrime) and expressed as the fractional abundance of isotopic fractions (^15^N/^14^N) multiplied by 100.

Concentration of vitamin B_12_ in samples was analyzed using a chemiluminescence solid-phase competitive assay at Applied Bioscience, LLC, Texas. Prior to analysis, samples underwent a series of preparatory steps to ensure accuracy and consistency. First, the samples were centrifuged at 2,500 rpm for 15 min at 4 °C to separate the supernatant from solid components. The resulting supernatant was carefully transferred into 12 × 75 mm polystyrene Falcon tubes with snap caps and stored at a temperature between 4 and 8 °C to preserve sample integrity. To prepare for filtration, the supernatant was aspirated using a 3 ml BD syringe equipped with a 20-gauge needle. The needle was then removed and replaced with a 0.45 µm syringe filter, allowing for effective removal of any remaining particulates. The filtered fluid was transferred into a new Falcon tube. If immediate analysis was not possible, the samples were stored at –20 °C until further use. In order to align with the linear detection range of the assay, a 1:10 dilution of the samples was performed using sterile phosphate-buffered saline (PBS). The samples were then assayed using the chemiluminescence method, which is a solid-phase competitive immunoassay technique. The assay’s sensitivity ranged from 125 pg/mL to 1000 pg/mL, corresponding to the linear portion of the standard curve.

### Statistical analyses

Data of daily average pH, NH_3_-N, VFA, lactate, and degradability of DM, OM, CP, NDF, N metabolism, and vitamin B_12_ were analyzed as a replicated 4 × 4 Latin square design using the MIXED procedure of SAS 9.4 (SAS Institute Inc., Cary, NC, United States). Fermenter within square was considered as the experimental unit. The model included the fixed effect of treatment and the random effects of fermenter within square, period, square, and day. Significance threshold was defined at *P *≤ 0.05, while 0.05 < *P *≤ 0.10 was considered a tendency. Multiple comparisons between treatments were evaluated with Tukey’s test.

## Results and discussion

### pH of ruminal contents

Ruminal pH is a crucial factor for optimum rumen function due to its effect on rumen microorganisms and fermentation (Gozho et al., 2006; Chibisa et al., 2016). In our continuous culture experiment (Table 2), we observed an average 6.19 pH across treatments, indicating that the ruminal pH was influenced similarly by all Co sources tested (*P *= 0.92).

**Table 2.** Effects of different cobalt sources on microbial ruminal fermentation metabolites of effluents in a dual-flow continuous-culture system.

Items^b^	**Treatments** ^a^				SEM	*P*-value
	CoCO_3_	CoPec	CoAA	CoPec+CoAA		
**pH**	6.16	6.17	6.18	6.24	0.03	0.92
**NH_3_-N, mg/dL**	17.2	15.1	18.9	14.0	3.65	0.41
**Lactate, mM**	0.21^a^	0.16^a^ ^b^	0.15^b^	0.14^b^	0.03	0.04
**D-lactate, mM**	0.14^a^	0.10^b^	0.10^b^	0.09^b^	0.03	0.03
**L-lactate, mM**	0.07	0.07	0.06	0.05	0.01	0.31
**Total VFA, mM**	58.5	54.1	59.5	55.1	2.62	0.33
***VFA molar proportion***						
**Acetate**	53.3	53.4	53.9	53.8	0.72	0.90
**Propionate**	23.7	21.9	22.8	21.3	1.43	0.33
**Butyrate**	15.6	17.8	16.2	17.8	1.19	0.16
**Isobutyrate**	0.61	0.66	0.61	0.67	0.03	0.48
**Isovaleric**	2.78	2.45	2.69	2.46	0.29	0.65
**Valeric**	2.79	2.51	2.57	2.45	0.17	0.42
**Caproic**	1.01	1.32	0.89	1.52	0.48	0.11

a Experimental treatments: **CoCO_3_**, Cobalt Carbonate; **CoPec**, Co Pectin; **CoAA**, Co Amino Acid; **CoPec+CoAA**, Co Pectin + Co Amino Acid. Supplemental Co sources provided a total of 1 mg Co/kg DM.

b Items: A: P = Acetate: Propionate.

Means within a row with different subscripts (a and b) differ (*P *≤ 0.05).

In our study, all treatments consisted of the same basal diet, which means all fermenters received the same daily provision of fermentable substrates. Therefore, any difference in pH would be attributed to possible differences in ruminal fermentation products promoted by different Co sources. However, the average pH values observed did not differ among all treatments and were within the normal range expected for adequate cellulolytic activity and fiber degradation (Orskov and Ryle 1990), indicating that major fermentation conditions affecting pH were similar across treatments. Previous studies in continuous culture fermenters have reported effects on pH when dietary starch is increased, but similar pH values in response to microbial additives (Monteiro et al., 2022). This indicates the relatively low responsiveness of pH unless contrasting diets are evaluated.

### Lactate

Lactate concentration is usually related to starch fermentation and the activity of lactate-producing and lactate-utilizing bacteria (Golder and Lean 2024). Total lactate concentration (Table 2) was lower in CoAA and CoPec+CoAA compared to CoCO_3_ (*P *= 0.04). Moreover, L-lactate concentrations were similar among treatments in continuous culture (*P *= 0.31), therefore the differences in total lactate were influenced mostly by the effect of treatment on D-lactate (*P *= 0.03), where all organic Co sources (CoPec, CoAA, and CoPec+CoAA) had a lower D-lactate concentration than CoCO_3_.

The Co and vitamin B_12_ requirement for ruminal synthesis of propionate has been established for specific ruminal bacteria such as *Prevotella* and *Succinivibrioceae* that are involved in the succinate pathway of propionate synthesis (Strobel, 1992; Franco-Lopez et al., 2020). However, to the best of our knowledge, it has not been reported whether Co is required for the acrylate pathway of propionate synthesis from lactate. Based on our results, lower D-lactate concentration for the CoPec, CoAA and CoPec+CoAA treatments may indicate either lower synthesis or greater utilization of D-lactate by ruminal microorganisms when these Co sources are supplemented in place of CoCO_3_.

*Megasphaera elsdenii* are the main lactate-utilizing bacteria in the rumen, converting lactate into propionate through the acrylate pathway (Prabhu et al., 2012, Da Silva-Cabral and Weimer 2024), while *Selenomonas ruminantium* is primarily recognized for its role in propionate production via the succinate pathway (Firkins and Mitchell 2023) and some evidence suggests it may also play a role as a lactate utilizer (Callaway and Martin 1997) recognizing the existence of a lactilytic group of *Selenomonas ruminantium* (Firkins and Mitchell 2023). However, there is no evidence that the activity or abundance of these bacteria are influenced by Co supplementation. Nevertheless, *Selenomonas* has shown greater lactate utilization for propionate synthesis when supplemented with yeast cultures (Callaway and Martin 1997), which the authors attributed to the presence of soluble growth factors such as B vitamins in yeast cultures.

Our results suggest a possible role of Co on lactate utilization by ruminal bacteria leading to a lower concentration of D-lactate. Although both D and L isomers contribute to the decrease in ruminal pH, their rate of absorption through ruminal epithelium may differ, with it being greater for L-lactate. This contributes to D-lactate accumulation in the rumen which plays an important role in ruminal acidosis (Nagaraja and Titgemeyer 2007; Golder and Lean 2024). Therefore, a potential reduction in D-lactate concentration would be beneficial for ruminal fermentation and health. Further research is needed to elucidate the primary pathways of ruminal lactate utilization, the microbial populations involved, and the possible animal response in dairy cows supplemented with CoAA.

### VFA concentration

Daily average concentrations of VFA for the dual-flow continuous culture experiment are shown in Table 2. There was no effect of treatment on the total VFA concentration and molar proportions in the fermenters. The type of substrate fermented, the microbial species involved, and the rumen environment (ie, pH and redox potential) during fermentation are key factors that affect VFA production in the rumen (Griswold et al., 1996). Since our experimental diets had the same ingredient and nutrient composition, differing only in the supplemental source of Co, then any possible treatment effect on VFA would be attributed to Co source.

Co supplementation increases propionate synthesis by certain ruminal bacteria such as *Prevotella*, that are dependent on vitamin B_12_ to convert succinate to propionate (Strobel 1992). However, we did not observe any effect of Co supplementation on propionate molar proportion and acetate to propionate ratio in ruminal contents. Tiffany et al. (2006) found greater molar proportions of butyrate, valerate, and isovalerate when CoCO_3_ increased Co concentration from 0 to 1 mg/kg DM in continuous culture fermenters; however, propionate molar proportion was similar across treatments.

Previous studies have documented the importance of vitamin B_12_ for ruminal synthesis of propionate. A greater molar proportion of propionate in the rumen of Co-supplemented steers during the finishing phase may explain the greater plasma glucose concentrations observed in steers receiving supplemental Co (Chen and Wolin 1981; Tanner and Wolfe 1988; Strobel 1992). Studies evaluating the impact of Co supplementation levels on ruminal fermentation may allow for greater magnitude of Co treatment effects when compared to a negative control without Co. In our study, the fact that all treatments received an equivalent dose of supplemental Co may reduce the magnitude of differences among treatments and consequently decrease the chances of observing a treatment effect on ruminal propionate and VFA.

Our continuous culture results indicate that all the organic Co sources tested decreased D-lactate concentration compared to CoCO_3_, which suggests a greater utilization of lactate as a substrate by ruminal bacteria when organic Co sources were fed. However, the lack of response on VFA makes it difficult to identify the fate of D-lactate in our experiment. Some lactate utilizers are able to synthesize propionate, acetate, and butyrate from lactate (Da Silva-Cabral and Weimer 2024) which may have contributed to the challenging of observing clear responses on a specific VFA in our study.

### Nutrient degradability

Degradability of OM (*P *= 0.59), CP (*P *= 0.82), and starch (*P *= 0.51) by ruminal microorganisms was similar across the Co sources tested (Table 3). We found an effect of treatment on the NDF degradability, indicating that diets formulated with CoPec+CoAA and CoAA have a greater NDF degradability than CoCO_3_ and CoPec (*P *< 0.01). These results suggest that sources of Co that favored a lower total lactate concentration also promoted greater NDF degradability by ruminal microorganisms. Moreover, the NDF degradability was greater for CoAA as compared to CoPec and CoCO_3_. The NDF degradability for CoAA and CoPec+CoAA was almost 12 percentage units greater, averaging 47.3% as compared to 36.06% for CoCO_3_ and 34.84% for CoPec.

**Table 3.** Effects of different cobalt sources on ruminal degradability of nutrient fractions in a dual-flow continuous-culture system.

Items^b^	**Treatments** ^a^				SEM	*P*-value
	CoCO_3_	CoPec	CoAA	CoPec+CoAA		
**OM, %**	58.6	59.7	60.2	61.2	1.93	0.59
**CP, %**	47.9	48.6	46.5	50.0	3.40	0.82
**NDF, %**	36.1^b^	34.8^b^	46.6^a^	48.0^a^	3.89	<0.01
**Starch, %**	90.9	90.6	91.5	89.9	1.36	0.51

a Experimental treatments: **CoCO_3_**, Cobalt Carbonate; **CoPec**, Co Pectin; **CoAA**, Co Amino Acid; **CoPec+CoAA**, Co Pectin + Co Amino Acid. Supplemental Co sources provided a total of 1 mg Co/kg DM.

b OM = Organic Matter, CP = Crude Protein, NDF = Neutral Detergent Fiber. Expressed as a percentage of DM.

Means within a row with different subscripts (a and b) differ (*P *≤ 0.05).

Such an increase in NDF degradability suggests that better conditions for ruminal bacteria associated with fiber degradation occur when CoAA is fed as a supplemental source of Co, which also favors lower concentrations of lactate. Succinate utilizing bacteria such as *Selenomonas ruminantium* and *Succiniclasticum ruminis* prevent accumulation of succinate in the rumen by performing propionate synthesis through the succinate pathway, which in turn favors cellulolytic bacteria such as *Fibrobacter succinogenes* by preventing a thermodynamic inhibition of succinate synthesis (Firkins and Mitchell 2023). *Selenomonas ruminantium* does not stimulate fiber degradation when grown in monoculture, however, it stimulates fiber digestion when co-cultured with succinolytic bacterium *Fibrobacter succinogenes*, which in turn stimulates propionate synthesis (Sawanon et al., 2011).

In our experiment we did not measure succinate concentration of samples and did not find any effect of Co treatment on propionate molar proportion, therefore we cannot confirm any possible changes in succinate utilization that may interfere with NDF degradability. However, in our study we found that a lower lactate concentration is consistent with greater NDF degradability when CoAA is the main supplemental source of Co. Considering the existence of a lactilytic group of *Selenomonas ruminantium* (Firkins and Mitchell 2023) we could speculate that CoAA stimulates lactate utilization by this type of bacteria, which results in a decrease of lactate concentration and stimulates NDF degradability.

Hussein et al. (1994) evaluated the effect of Co supplementation on in vitro digestion by supplementing Co glucoheptonate at 0, 5 and 10 or at 0, 10, 20, and 30 mg of Co per kg of diet DM. At such high levels of supplementation, they did not find any improvements on in vitro DM or NDF degradability. In our experiment, not only is the supplemental dose of Co much lower, but also all diets received an equivalent dose of Co, indicating that differences in NDF degradability may be attributed to the effect of Co source.

Martinez and Church (1970) reported that Co had a stimulatory effect on in vitro cellulose digestion at concentrations of 3 mg/kg DM, while negative effects on cellulose digestion were reached at concentrations of 7 mg/kg DM. This suggests that lower levels of supplementation such as the one in our study (1 mg of supplementary Co per kg DM) may be beneficial for cellulolytic activity. Greater ruminal degradability of NDF can be very beneficial for dairy cows. Better utilization of NDF in the rumen has positive effects on DMI of cows which consequently improves milk production (Oba and Allen 1999b; Kendall et al., 2009).

Specifically, within the context of corn silage-based diets, there has been growing interest in finding strategies to increase NDF degradability of diets by improving quality of silage and genetic selection of plants such as brown mid rib hybrids. These strategies have been shown to have positive impacts on DMI and production (Oba and Allen 1999a). It has been estimated that a one unit increase in NDF degradability (including in vivo, in vitro, and in situ data) is associated with a 0.17 kg increase in DMI and 0.25 kg increase in energy corrected milk production (Oba and Allen 1999b). Therefore, strategies that improve NDF degradability of diets can be very advantageous for the dairy industry.

### Nitrogen metabolism

As presented in Table 4, Co source did not affect daily flows of NH_3_-N (*P *= 0.93), NAN (*P *= 0.22), and bacterial efficiency (*P *= 0.40). However, bacterial N flow (*P *= 0.01) and efficiency of N utilization (*P *= 0.01) were greater for CoAA as compared to CoCO_3_ and CoPec.

**Table 4.** Effects of different cobalt sources on N metabolism in a dual-flow continuous-culture system.

Items	**Treatments** ^a^				SEM	*P*-value
	CoCO_3_	CoPec	CoAA	CoPec+CoAA		
**Total N, g/d^b^**	3.22	3.19	3.43	3.24	0.12	0.32
**NH_3_-N, g/d^c^**	0.38	0.36	0.37	0.37	0.04	0.93
**NAN, g/d^d^**	2.84	2.83	3.06	2.87	0.14	0.22
**Bacteria N, g/d^e^**	1.42^b^	1.43^b^	1.61^a^	1.51^a^ ^b^	0.07	0.01
**Dietary N, g/d^f^**	1.42	1.40	1.45	1.36	0.09	0.82
**ENU, %^g^**	52.3^b^	52.7^b^	59.4^a^	55.7^a^ ^b^	2.71	0.01
**Bacterial efficiency, g/kg^h^**	25.0	24.4	26.4	25.3	1.63	0.40

a Experimental treatments: **CoCO_3_**, Cobalt Carbonate; **CoPec**, Co Pectin; **CoAA**, Co Amino Acid; **CoPec+CoAA**, Co Pectin + Co Amino Acid. Supplemental Co sources provided a total of 1 mg Co/kg DM.

b Total N = total N flow (g/d) = NH_3_-N + NAN (Bach and Stern 1999).

c NH_3_-N = ammonia N flow (g/d) = mg/dL of effluent NH_3_-N × (g of total effluent flow/100).

d NAN = nonammonia N flow (g/d) = total N − NH_3_-N.

e Bacterial-N flow (g/d) = (NAN flow × % atom excess of 15 N in NAN effluent)/(% atom excess of 15 N in bacteria pellet), according to Calsamiglia et al. (1996).

f Dietary N flow (g/d) = g of effluent NAN − g of effluent bacterial N.

g ENU = efficiency of N use = (g of bacterial N/g of available N) × 100 (Bach and Stern 1999).

h Bacterial efficiency = g of bacterial N flow/kg of OM truly digested (Calsamiglia et al., 1996).

Means within a row with different subscripts (a and b) differ (*P *≤ 0.05).

One could speculate that favoring lactate utilization through CoAA supplementation, which presumably favors fiber degrading bacteria and therefore increases NDF degradability, may increase NH_3_-N utilization by cellulolytic bacteria, resulting in greater efficiency of N utilization and bacterial N flow. However, in our experiment we did not find any effect of Co source on NH_3_-N. Similarly, Tiffany et al. (2006) reported that supplementing CoCO_3_ at 0, 0.05, 0.1, and 1.0 mg of Co/kg DM did not affect concentrations of propionate and NH_3_-N in continuous culture fermenters.

Greater bacterial N flow and efficiency of N utilization in CoAA as compared to CoCO_3_ are indicative of a better ruminal environment for bacteria when Co is supplemented as CoAA, which is consistent with a lower D-lactate concentration when diets included CoAA as a supplemental source of Co. Lactate utilization is recognized as one of the main mechanisms that some ruminal bacteria (particularly *Megasphaera* and some *Selenomonas*) use as a strategy to adapt to greater ruminal concentrations of lactate and maintain efficiency of microbial protein synthesis (Hackmann and Firkins 2015). This supports a possible explanation for the positive effects on bacterial N flow observed in our study when CoAA was included in the diet, suggesting a greater microbial protein synthesis. Microbial analysis would allow a better understanding of the effects of Co sources on specific microbial populations, which would be a very good complement to the data on VFA, lactate, and N metabolism; however, we do not have samples for microbial analysis in this study, so this can be considered for future evaluations.

### Vitamin B_12_ metabolism

Effects of experimental Co sources on vitamin B_12_ metabolism are summarized in Table 5. We measured several outputs of vitamin B_12_ in the dual flow continuous culture, and most of them were similar among the Co sources evaluated, such as B_12_ flow (*P *= 0.95), B_12_ efficiency (*P *= 0.97), liquid associated B_12_ efficiency (*P *= 0.13), particle associated B_12_ flow (*P *= 0.91), and particle associated B_12_ flow efficiency (*P *= 0.83). However, we found that the flow of vitamin B_12_ associated with the liquid effluent tended to be lower for CoCO_3_ as compared to CoPec, CoAA, and CoPec+CoAA (*P *= 0.10).

**Table 5.** Effects of different cobalt sources on B_12_ metabolism in a dual-flow continuous-culture system.

Items	**Treatments** ^a^				SEM	*P*-value
	CoCO_3_	CoPec	CoAA	CoPec+CoAA		
**B_12_ flow, µg/d^b^**	4.95	5.04	4.75	4.94	0.41	0.95
**B_12_ efficiency, µg/kg^c^**	68.2	68.6	65.2	66.8	5.82	0.97
**LA B_12_ flow, µg/d^d^**	2.85	3.69	3.21	3.05	0.34	0.10
**LA B_12_ efficiency, µg/kg^e^**	39.4	49.6	44.3	41.2	4.64	0.13
**PA B_12_ flow, µg/d^f^**	5.42	5.39	5.27	4.97	0.49	0.91
**PA B_12_ efficiency, µg/kg^g^**	74.5	73.1	72.5	66.5	6.91	0.83
**B_12_ flow SL, µg/d^h^**	8.27	8.99	8.46	8.02	0.70	0.74

a Experimental treatments: **CoCO_3_**, Cobalt Carbonate; **CoPec**, Co Pectin; **CoAA**, Co Amino Acid; **CoPec+CoAA**, Co Pectin + Co Amino Acid. Supplemental Co sources provided a total of 1 mg Co/kg DM.

b B_12_ flow (µg/d) = [(Liquid flow (mL/d) + Solid flow (mL/d)) × pooled B_12_ (pg/mL)]/1,000,000.

c B_12_ pool efficiency (µg/kg) = B_12_ flow (µg/d)/DMD (kg/d).

d Liquid Associated B_12_ flow (µg/d) = Liquid flow (mL/d) × LA B_12_ (pg/mL)/1,000,000.

e Liquid Associated B_12_ efficiency (µg/kg) = Liquid Associated B_12_ flow (µg/d)/DMD (kg/d).

f Particle Associated B_12_ flow (µg/d) = Solid flow (mL/d) × PA B_12_ (pg/mL)/1,000,000.

g Particle Associated B_12_ efficiency (µg/kg) = Particle Associated B_12_ flow (µg/d)/DMD (kg/d).

h Total flow of B_12_ (µg/d) = Liquid Associated B_12_ flow (µg/d) + Particle Associated B_12_ flow (µg/d).

These results suggest that the organic Co sources tested in our experiment may have greater availability in the rumen, consequently improving vitamin B_12_ synthesis by ruminal bacteria. Conversely, flow of vitamin B_12_ associated with solid particles was similar among tested Co sources, indicating that improved Co availability of CoPec, CoAA, and CoPec+CoAA, primarily impacts the liquid fraction of ruminal contents. Kawashima et al. (1997) evaluated Co sources (sulfate, carbonate, oxide, and glucoheptonate) at 1 and 40 mg/kg in semi-continuous culture of ruminal contents, and found that cultures supplemented with Co sulfate, CoCO_3_, and Co glucoheptonate had greater vitamin B_12_ synthesis compared to Co oxide, suggesting possible source effects on ruminal utilization of Co, indicating a possibly lower ruminal solubility and availability of Co oxide compared to the other sources.

Increasing CoCO_3_ supplementation from 0 to 1 mg of Co/kg DM promoted a four-fold increase in vitamin B_12_ concentrations in continuous culture fermenters (Tiffany et al., 2006). Other studies have confirmed that Co supplementation increased vitamin B_12_ status in lactating dairy cows supplemented with 25 or 75 mg of Co from either CoCO_3_ or Co glucoheptonate as reflected in liver and milk vitamin B_12_ concentrations (Akins et al., 2013), This indicates that increasing Co supplementation can also increase ruminal synthesis and further absorption of vitamin B_12_. All the treatments in our study provided the same amount of supplemental Co, therefore, a greater vitamin B_12_ flow in liquid effluent suggests that a greater amount of Co was available when CoPec, CoAA, and CoPec+CoAA were fed, in comparison to CoCO_3_.

## Conclusions

Our results indicate that under the conditions of this experiment, corn silage-based diets formulated with ground corn, soybean meal, cotton seed, citrus pulp, and 1 mg of supplemental Co/kg DM from CoPec, CoAA, and CoPec+CoAA had greater NDF degradability and increased vitamin B_12_ flow in the liquid fraction compared to diets containing CoCO_3_. ­Additionally, diets formulated with CoAA, alone or combined with CoPec, showed reduced D-lactate concentrations and greater microbial protein flow. These in vitro findings suggest that greater NDF degradability and vitamin B_12_ flow may be associated with greater lactate utilization by ruminal bacteria when CoAA is used as the Co source in the diet; however, there were no differences in propionate synthesis among the Co sources tested.

## LIST OF ABBREVIATIONS:

(^15^NH_4_)_2_SO_4_: nitrogen 15 labelled ammonium sulfate
^15^N: nitrogen 15 isotope
Co: cobalt
CoAA: cobalt amino acid
CoCO_3_: cobalt carbonate
CoPec: cobalt pectin
CP: Crude Protein
DM: Dry Matter
DMI: Dry matter intake
ENU: Efficiency of nitrogen utilization
K2CO3: potassium carbonate
N: Nitrogen
NAN: Non ammonia nitrogen
NDF: Neutral Detergent Fiber
NH_3_-N: Ammonia nitrogen
OM: Organic Matter
RDP: Rumen Degradable Protein
RUP: Rumen Undegradable Protein
VFA: Volatile Fatty Acids
